# Dr. Frederick H. Parker

**Published:** 1918-07

**Authors:** 


**Dr. Frederick H. Parker** of Auburn died recently, age 62. He graduated from the New York University, Medical Department, in 1881.

